# A^3^C-TL-GTO: Alzheimer Automatic Accurate Classification Using Transfer Learning and Artificial Gorilla Troops Optimizer

**DOI:** 10.3390/s22114250

**Published:** 2022-06-02

**Authors:** Nadiah A. Baghdadi, Amer Malki, Hossam Magdy Balaha, Mahmoud Badawy, Mostafa Elhosseini

**Affiliations:** 1 College of Nursing, Princess Nourah Bint Abdulrahman University, Riyadh 11671, Saudi Arabia; nabaghdadi@pnu.edu.sa; 2 College of Computer Science and Engineering, Taibah University, Yanbu 46421, Saudi Arabia; asamalki@taibahu.edu.sa (A.M.); melhosseini@mans.edu.eg (M.E.); 3 Computers and Control Systems Engineering Department, Faculty of Engineering, Mansoura University, Mansoura 35516, Egypt; hossam.m.balaha@mans.edu.eg

**Keywords:** Alzheimer, artificial gorilla troops optimizer (GTO), convolutional neural network (CNN), deep learning (DL), metaheuristic optimization

## Abstract

Alzheimer’s disease (AD) is a chronic disease that affects the elderly. There are many different types of dementia, but Alzheimer’s disease is one of the leading causes of death. AD is a chronic brain disorder that leads to problems with language, disorientation, mood swings, bodily functions, memory loss, cognitive decline, mood or personality changes, and ultimately death due to dementia. Unfortunately, no cure has yet been developed for it, and it has no known causes. Clinically, imaging tools can aid in the diagnosis, and deep learning has recently emerged as an important component of these tools. Deep learning requires little or no image preprocessing and can infer an optimal data representation from raw images without prior feature selection. As a result, they produce a more objective and less biased process. The performance of a convolutional neural network (CNN) is primarily affected by the hyperparameters chosen and the dataset used. A deep learning model for classifying Alzheimer’s patients has been developed using transfer learning and optimized by Gorilla Troops for early diagnosis. This study proposes the A${}^{3}$C-TL-GTO framework for MRI image classification and AD detection. The A${}^{3}$C-TL-GTO is an empirical quantitative framework for accurate and automatic AD classification, developed and evaluated with the Alzheimer’s Dataset (four classes of images) and the Alzheimer’s Disease Neuroimaging Initiative (ADNI). The proposed framework reduces the bias and variability of preprocessing steps and hyperparameters optimization to the classifier model and dataset used. Our strategy, evaluated on MRIs, is easily adaptable to other imaging methods. According to our findings, the proposed framework was an excellent instrument for this task, with a significant potential advantage for patient care. The ADNI dataset, an online dataset on Alzheimer’s disease, was used to obtain magnetic resonance imaging (MR) brain images. The experimental results demonstrate that the proposed framework achieves 96.65% accuracy for the Alzheimer’s Dataset and 96.25% accuracy for the ADNI dataset. Moreover, a better performance in terms of accuracy is demonstrated over other state-of-the-art approaches.

## 1. Introduction

The prevalence of age-related diseases rises as people live longer, especially brain diseases, mostly neurodegenerative, such as Alzheimer’s disease (AD) [1]. AD was named in 1907 by Alois Alzheimer, who delineated a fifty-year-old woman dying of advanced dementia after four years of rapid memory deterioration [2]. AD is an irreversible, progressive, and ultimately fatal brain degenerative disorder that affects middle-aged and older people. When the disease is discovered, most patients have already progressed to an advanced stage [3]. As a result, AD gradually deteriorates memory and thinking abilities and the ability to carry out even the most basic duties of daily life by destroying the brain cells. Unfortunately, there is no currently available curative treatment for AD. Thus, early detection can effectively treat cognitive losses at the initial stage.

**Figure 1:**
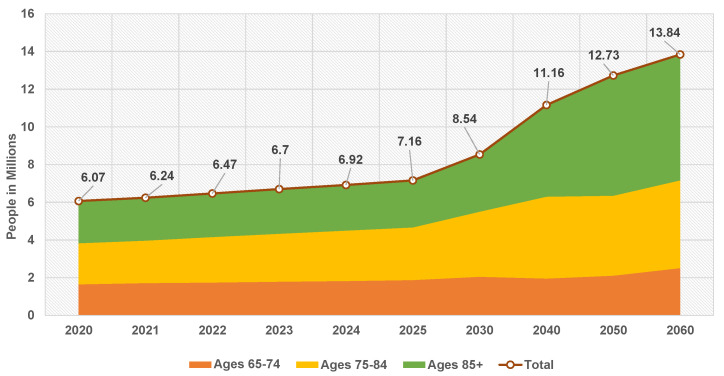
The anticipated number of US people above 65 with AD from 2020 to 2060.

Various ailments are associated with aging, and AD is a major cause of dementia, also known as a major neurocognitive disorder, which mainly affects older people and poses the highest cost to society and healthcare budgets. The estimated annual cost of dementia is one trillion dollars, and it is expected to double by 2030 [4]. The World Health Organization (WHO) stated that dementia is a major societal concern, with more than 55 million people worldwide suffering from dementia, with nearly 10 million new cases diagnosed each year and 82 million cases in the next ten years [5]. Furthermore, the report [6] pointed out that, by 2050, patients with dementia will reach 152 million, with a patient being diagnosed with dementia every three seconds [7]. AD is a progressively developing disease and is considered the seventh leading cause of death in the USA, with 132,741 deaths in 2020 [8], which exceeds breast and prostate cancer combined [9]. In addition, AD with unknown causes endangers the physical health of the elderly [7]. The aging of the world’s population is increasing year by year [3]. For the first time in US history, the speedup of global aging will outnumber children (77 million) by 2034. As a result, the incidence of AD will increase dramatically and become more challenging with this quickening of global population aging. Figure 1 reports the anticipated number of people above 65 with AD in the US population from 2020 to 2060 [10].

There are no viable therapy techniques or medications available for Alzheimer’s disease at the moment. Therefore, the diagnosis of dementia journey is often complex and experiences long wait times [11]. On the other hand, AD treatments at early stages slow down the complications and maintain the residual brain functions. Therefore, early detection and intervention for this central nervous system degeneration are crucial to providing timely treatment to patients. In this vein, a complete understanding of its biomarkers is essential to differentiate AD symptoms from normal aging symptoms and accordingly slow its progression. Indeed, many neurological disorders directly impact the brain, particularly the hippocampus, which is essential in forming memories [12], emotional control, and learning. Hippocampus damage has been linked to various neurological and psychiatric disorders, including AD [12]. Prolonged AD is linked to tissue loss in various brain regions [13]. The damage begins in the gray matter (GM) and progresses to the white matter (WM) before reaching the hippocampus [12].

**Figure 2:**
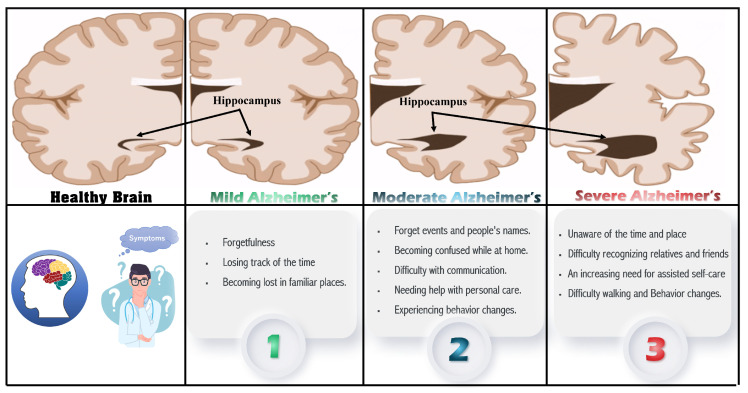
The major signs and symptoms of dementia.

Figure 2 shows the major signs and symptoms of dementia [5,11] that start with memory loss and end with death. Early on, AD manifests as a mild cognitive impairment (MCI) and gradually gets worse. MCI is a condition in which people have more memory problems than usual and increases the risk of developing AD in some older people than others. Mild Alzheimer’s patients are frequently diagnosed with getting lost, difficulty performing tasks, repeating questions, and behavioral changes. The disease progresses in stages, ranging from a moderate to severe AD stage [14]. Damage occurs in areas of the brain that control language, reasoning, and thought in the moderate AD stage. As a result, memory loss worsens, and people have difficulty recognizing others. Severe Alzheimer’s disease is distinguished by significant brain tissue shrinkage and plaques and tangles spread throughout the brain. Patients in this stage cannot communicate and must rely entirely on others for their care.

The manual diagnosis of AD is based on recent developments in advanced neuroimaging techniques (such as magnetic resonance imaging (MRI), Computed Tomography (CT), Positron Emission Tomography (PET)), manual feature extraction, and clinical evaluations. MRI scans are the commonly utilized method that achieved unprecedented progress due to their non-invasive nature, high resolution, nonionizing radiation, and multidirectional imaging [15]. However, the brain structure is very complicated, and the imaging modalities involved are multi-modal and the curse of dimensionality, making the manual diagnosis time-consuming, error-prone, and tedious.

Recently, the rise of decision support systems based on medical imaging analysis has a great role in developing intelligent diagnosis systems for AD that can identify the severity of the patient’s disease and, therefore, keep AD in the initial stage. Furthermore, artificial intelligence and machine learning appear to be promising solutions that aid radiologists in an AD diagnosis. Thus, the accurate classification approach of brain images in the different stages of the disease can be efficiently performed. However, the AD diagnosis based on traditional machine learning algorithms had different time and space complexity, statistical data distribution, convergence, and overfitting challenges. Deep learning (DL) has recently been used in image classification to solve these challenges and introduced an accurate medical image classification approach. The key elements of a successful DL model are: the used datasets for training and testing, the design of the network, and the parameters and hyperparameter optimization [16]. Current deep learning approaches are effective in medical image evaluation as they do not require great effort for prior preprocessing and feature selection, resulting in a more objective and less biased process [17]. As a result, deep learning can efficiently classify brain images at various stages of the disease.

The main objective of this study is to propose an A${}^{3}$C-TL-GTO framework for MRI image classification and Alzheimer’s disease detection. The proposed framework consists of four phases: (1) Acquisition Phase, (2) Preprocessing Phase, (3) Classification, Learning, and Optimization Phase, and (4) Population Updating Phase. The A${}^{3}$C-TL-GTO framework is based on transfer learning and the Artificial Gorilla Troops Optimizer (GTO). The main contributions of this study are:

- Introduce a novel Alzheimer classification framework based on pretrained CNNs.
- A CNN architecture is chosen based on an analysis of an Alzheimer’s patient brain MRI scans formulated as an optimization problem handled by Gorilla Troops Optimizers on the list of top algorithms that outperform natural-inspired algorithms.
- The performance of each pretrained model is improved by optimizing a CNN and transfer learning hyperparameters with Gorilla.
- There is no need to manually configure hyperparameters because this framework is adaptable.
- The findings of standard performance measurements have been quite promising.

The paper is organized as follows: The background is introduced in Section 2. In Section 3, related work is reviewed. Section 4 describes the proposed A${}^{3}$C-TL-GTO framework and algorithms. Section 5 discusses the experiments and the results. Section 6 concludes the paper.

## 2. Background

**Figure 3:**
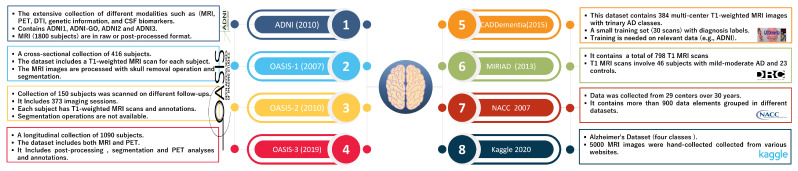
The characteristics of well-known AD datasets.

Alzheimer’s disease (AD) is a type of dementia that progresses over time and is among the many ailments associated with aging. Alzheimer’s disease develops gradually over the years, and there is no cure. However, older people are more prone to AD. Early-onset is rare [18]. However, AD is fatal if left untreated. Diagnosing AD at an early stage is imperative because existing treatments only slow the progression of symptoms [12,16]. One of the neurologists’ most difficult issues is classifying Alzheimer’s disease (AD). Methods using manual classification can be time-consuming and inaccurate. Because the brain is the most impacted region in AD [19], a precise classification framework based on a brain imaging dataset may deliver better results. Various research studies use different datasets to evaluate and compare their proposed methodology with other state-of-the-art research [2]; Figure 3 summarizes the characteristics of well-known AD datasets. Historically, basic scientific findings concerning neurological disorders have been hard to translate into effective treatments.

Nevertheless, gathering and manipulating large datasets has become exponentially easier with big data. Multi-modal and multidimensional datasets, such as imaging and genomics analysis, are among these complicated datasets. Analytics become more challenging as datasets grow. Advanced statistical and mathematical algorithms are being used to tackle this formidable challenge based on machine learning, deep learning, and deep reinforcement learning. Computer-aided techniques and medical imaging are the most reliable means of detecting AD early [20,21]. In recent years, deep learning has received great success in the medical image field. As well as being used in medical image analysis, it has also gained wide attention for AD detection [22].

The AI learning model learns directly from the data, and as it is exposed to huge datasets and trained over time, it gets better. The model can make predictions based on previously unknown data with this knowledge. AI learning models are classified into three types: a supervised model for structured and labeled data, an unsupervised model for unlabeled and unstructured data, and a semi-supervised model, which combines both. Machine learning techniques, such as deep learning (DL), simulate the brain’s functions to create patterns and identify patterns that can be used to make more complex decisions. DL is the first choice for researchers because of its ability to draw information from even unstructured and unlabeled data [19]. In DL calculations, many nonlinear layers can be used to extract features. Each layer contributes to the depth of understanding of a system. A DL family member, a convolutional neural network (CNN), typically analyzes images without prior processing [21]. For identifying documents [23], LeCun and others introduced a deep CNN in 1998. Machine learning has been used as a diagnostic tool by physicians in recent years, as it offers additional information [20,23].

Deep learning is predicted to be the future of artificial intelligence, but it requires enormous amounts of data. When feature spaces change, algorithms must be rebuilt to address new problems. In previous studies, the network was generally built from the ground up, which is rarely achievable, and the training process is time-consuming, labor-intensive, and ineffective. Because transfer learning is much faster and more effective than traditional learning, using pretrained networks, such as AlexNet, to identify images changed the significance of DL networks in the long term. Furthermore, this is inappropriate for small radiology datasets, and overfitting is prevalent during training [18,24]. Deep learning layers transferred between datasets could be an interesting research topic for various tasks. Meta-learning may achieve higher reuse levels in the future. Despite the difficulty of the process, researchers can use a variety of internet databases and software packages to identify AD. The depth model can be implemented using Matlab, Keras, Tensorflow, Torch, and other software programs.

Because deep learning models outperform traditional models on large datasets, the methods described above are less reliable when applied to clinical cases. In addition, the models above depend on standard parameters. The chosen hyperparameters and datasets [25] significantly influence the CNN performance. Hyperparameters are different from model weights. The former is determined before training, whereas the latter is determined during training. Hyperparameters can be adjusted in several ways [25]. A poor choice of hyperparameters can negatively impact the performance of an application [26]. Therefore, hyperparameter values are selected according to an optimization process [25] instead of being randomly selected for each application.

A proposed framework will typically include numerous layers, intermediate processing elements, and other structural features, necessitating the use of search metaheuristics to find these hyperparameters. The metaheuristic algorithm provides accurate and robust solutions to nonlinear, multidimensional optimization problems. Most metaheuristics derived from natural organisms in nature are used to solve optimization problems [27]. Furthermore, because metaheuristics use a black-box approach, they have high flexibility and no gradient information, making them simple to use and not reliant on gradient information. Regardless of structural characteristics, metaheuristic methods begin with random trial solutions within their limitations. The algorithm-specific equations then iteratively evolve candidate solutions until the termination condition is satisfied. As a result, various optimization algorithms can propose varying degrees of solution improvement [28]. Evolution, physics, and swarms are three commonly used metaheuristic algorithm types [29]. The swarm algorithm simulates a population’s social behavior. Since the early 1990s, various swarm-based optimization algorithms, such as particle swarm (PSO) and ant colony (ACO), have been developed. Swarm intelligence algorithms include firefly, grey wolf, sparrow, whale optimization, and artificial bee colony algorithms.

The Artificial Gorilla Troops Optimizer is a new algorithm based on gorilla natural behaviors (GTO). In 2021, Abdollahzadeh et al. proposed the GTO. Gorillas’ social behavior and movement are mimicked in this method [27,30]. Troops of gorillas consist of a silverback gorilla group and several females and their offspring. Male gorilla groups are also present. The silver hair that emerges on the silverback’s back during puberty gives it its name [27,31]. It has a lifespan of about 12 years. Therefore, a group’s attention is drawn to the silverback. However, it is not just the one who makes all the decisions but mediates fights, determines gorilla group movement, guides them to food sources, and is responsible for their safety and well-being. Blackbacks are young male gorillas who serve as backup guardians for silverbacks. They are between the ages of 8 and 12, and their backs are free of silver hairs. Gorillas, both male and female, move from their birthplaces. Normally, gorillas migrate to new groups. On the other hand, a male gorilla is more likely to abandon his group and start a new one by wooing a female gorilla who has traveled outside. Male gorillas may stay in the same group, although they were born silverbacks and are categorized as such. If the silverback dies, certain gorillas may strive to dominate the group or stand and fight with the silverback to attain their objectives [32].

The accuracy and efficiency of the GTO were demonstrated [31]. The optimizer is simple for engineering applications and does not require many adjustments [27]. Furthermore, the GTO algorithm can produce good results for a wide range of system dimensions by increasing search capabilities. Other optimizers’ performance drop significantly as the dimensions increase, giving them a significant advantage in all comparable dimensions [32]. For example, gorillas cannot live alone due to their group-living preferences. As a result, gorillas hunt for food in groups and are led by a silverback leader in charge of group choices. A silverback is regarded as the best in this algorithm, and any candidate of the gorillas tends to approach it. The weakest gorilla is excluded because it is the worst.

**Figure 4:**
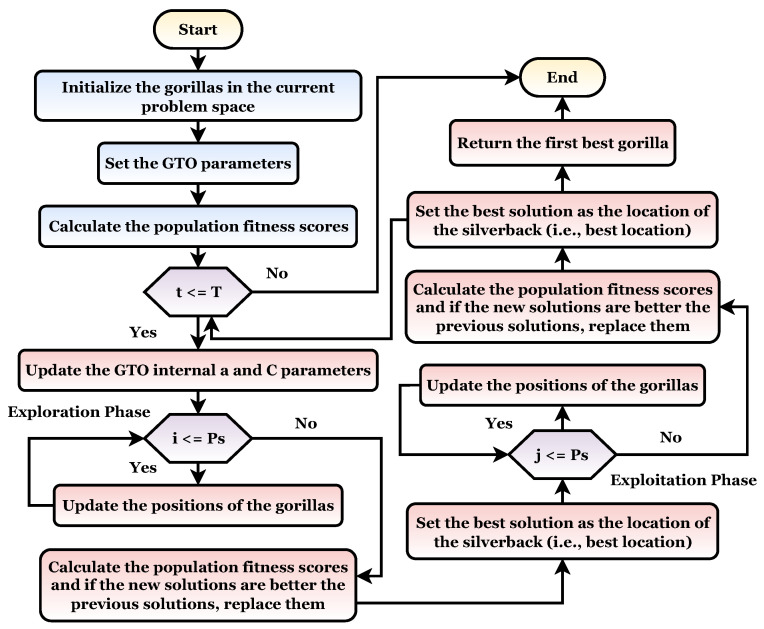
The gorilla natural behaviors flowchart.

In this algorithm, gorillas are denoted as *X*, while silverbacks are denoted as GX. For example, consider a gorilla on the hunt for better food sources. As a result, the iteration process generates GX each time and exchanges it with another solution if a better value can be determined [30]. The GTO flowchart is shown in Figure 4. This algorithm is also divided into two phases as follows.

### 2.1. Exploration Phase

Silverback gorillas are the best possible choice solutions for each optimization step in the GTO algorithm, and all gorillas are regarded as potential solutions. Exploration has been carried out with three operators: the movement to unknown places to expand the GTO exploration further. The second operator balances the gorilla exploration and exploitation by moving to other gorillas. With the third operator migrating toward a known site in the exploration phase, the GTO may explore different optimization spaces more effectively.

The migration mechanism was selected by using a parameter named *p*. Before conducting the optimization procedure [30], the factor (*p*) in the range 0–1 must be specified to determine the likelihood of adopting a migration strategy to an unidentified location. A first mechanism is selected when rand<p. However, if rand⩾0.5, the mechanism of approaching other gorillas is chosen. However, if rand<0.5, a movement to a well-known site is chosen, and each can deliver a good performance to the algorithm based on the strategies used. At the end of the exploration phase, all of the results are evaluated, and if GX(t) is the least expensive option, GX(t) is used instead of X(t) (Silverback). In addition, Equations (7)–(9) in Section 4.3.3 summarize three different mechanisms [27].

### 2.2. Exploitation Phase

There are two types of mechanisms for use during this phase. The first mechanism is “follow the silverback”, while the second includes “adult female competition”. The decision can be made by comparing the value of *D* with the random number W chosen at the start of the optimization procedure [27].

The newly established group’s leader Silverback is a young and fit gorilla whom the other male gorillas closely follow. Similarly, they follow Silverback’s orders to find food and travel to various locations. Members of a group can also influence each other’s movements within the group. For example, Silverback directs his gorillas to travel to food-supply locations to locate food, and this strategy can also be used with D⩾W. When young gorillas reach maturity, they struggle with other adult gorillas for the right to choose females for their group, which is a frequently violent process. This strategy can also be used when D<W. If GX(t) has a lower cost than X(t), GX(t) replaces X(t) and is found to be the best alternative (Silverback) [30].

## 3. Related Studies

Recently, many researchers studied machine learning in the medical field. Finding a more accurate and efficient method for diagnosing and predicting AD is a hot research topic [14]. Deep learning has great potential in diagnosing AD based on imaging or molecular data. This section explores the current state of the art that uses deep learning architectures for AD diagnosis and prediction.

Islam and Zhang [33] developed a DCNN model for AD four-class classification based on MRI images. The Inception-V4 model was trained and tested on the OASIS dataset. The proposed model achieved an accuracy of 73.75%. However, the proposed model suffered limited datasets and low accuracy. Zhang et al. [34] introduced an extreme learning machine (ELM) model for binary AD classification. First, manually segmented Voxel-based Morphometry images from the ADNI database of 627 patients were used. Then, feature calculation, simple feature extraction, and classification were performed using the ELM model. Ten-fold cross-validation was performed to ensure the ELM model validity, which achieved an accuracy of 96%. However, its major drawbacks are dataset limitation and poor feature selection.

Martinez et al. [35] studied applying deep learning to discover the relationship between symptoms, tests, and features extraction using Convolutional Autoencoders (CAEs). This study began with data acquisition from three data sources: MRI from the ADNI database, data obtained via the Alzheimer’s Disease Assessment Scale (ADAS), and the Clinical Dementia Ratio (CDR-SB). After data preprocessing, CAEs were used for feature extraction and manifold modeling and achieved a classification accuracy of 85%. Saratxaga et al. [2] developed an approach for the AD multi-class classification based on deep learning-based techniques. They used 305 MRI images from the OASIS database and CDR clinical annotation. They used different pretrained architectures, and the ResNet achieved the best results with an accuracy of 93%. Raees et al. [36] introduced a light DL classification and feature extraction approach. They deployed different pretrained models to build a trinary classifier. Functional MRI (fMRI) images retrieved from the ADNI database were used for training and testing. The VGG19 achieved the highest accuracy of 90%. Buvaneswari and Gayathri [37] introduced a segmentation, feature extraction, and classification approach based on deep learning. From the ADNI, 240 sMRI images with SegNet were used to train the ResNet-10 architecture for classification. The proposed approach recorded an accuracy of 95%.

Katabathula et al. [38] developed a lightweight 3D DenseCNN2 model for AD classification. The DenseCNN2 was built on the global shape and visual hippocampus segmentation. Their proposed model was trained and tested with 933 sMRI images obtained from the ADNI. The DenseCNN2 model achieved a classification accuracy of 92.52%. Mahendran and Vincent [12] developed a feature selection and classification approach for AD. They used a DNA methylation dataset that consisted of 68 records. First, preprocessing was performed to improve the classification performance. The feature selection was then applied using Ada Boost, Random Forest, and SVM to select useful genes. An Enhanced Deep Recurrent Neural Network (EDRNN) model was used for classification. They used the Bayesian optimization technique with five-fold cross-validation for hyperparameter optimization. The approach achieved an accuracy of 87% with the Ada Boost. Zhang et al. [39] introduced an effective CNN-based framework based on T1-weighted structural MRI (sMRI) images from the ADNI. Data preprocessing was performed using conventional procedures. An improved framework tresnet of a residual network was used for classification. The proposed method achieved a classification accuracy of 90%.

Liu et al. [15] developed a multi-scale CNN with a channel attention mechanism for enhanced AD diagnosis. They used preprocessing and segmentation to obtain the WM and GM datasets and model training. They extracted multi-scale features and fused them between channels to obtain more comprehensive information. ResNet-50 was used and achieved an accuracy of 92.59%. The CLSIndRNN model for AD feature selection and classification was introduced in [9] using the ADNI dataset, which contains 805 samples of MRI images. A recurrent neural network regression was used to predict the early diagnosis clinical score. Image preprocessing, feature selection, and classification techniques proved the effectiveness of the proposed model in clinical scores prediction. Shanmugam et al. [16] introduced a transfer learning-based approach for multi-class detection for cognitive impairment stages and AD. They used GoogLeNet, AlexNet, and ResNet-18 networks trained and tested by 6000 MRI ADNI images. The ResNet-18 network achieved the highest classification accuracy of 98.63%. Kong et al. [3] developed a deep learning-based strategy that involved a novel MRI and PET image fusion and 3D CNN for AD multi-classification methods. The ADNI dataset of 740 3D images was used. The proposed strategy achieved an accuracy of 93.5%. A study [40] applied network architecture and hyperparameters optimization based on a Genetic Algorithm. They used an amyloid brain image dataset that contains PET/CT images of 414 patients. The proposed algorithm achieved a classification accuracy of 81.74%. A TL-based approach for Alzheimer’s diagnosis based on sagittal MRI (sMRI) was introduced in [13]. The authors used the ADNI and OASIS datasets and concluded that sMRI can be used effectively to differentiate AD stages and that TL is necessary for completing the task.

Helaly et al. [4] developed a deep learning-based framework for the early multi-classification of AD named the $E^{2}AD^{2}C$ framework. The $E^{2}AD^{2}C$ framework consists of six stages: Data Acquisition, Preprocessing, Data Augmentation, Classification, Evaluation, and Application. For classification, they used two architectures: (1) the light CNN architectures and (2) transfer learning-based architecture. The ADNI dataset for 300 patients divided into four classes was used. The E2AD2C framework achieved accuracies of 93.61% and 95.17% for 2D and 3D multi-class AD stage classifications. In addition, an accuracy of 97% was recorded via the VGG19 model. Then, the same authors developed a deep learning-based framework for hippocampus segmentation [41] using the U-Net architecture. This framework consists of four stages: data acquisition, preprocessing, data augmentation, and segmentation. The segmentation step was performed via two architectures: (1) the U-Net architecture with hyperparameter tuning and ResNet pretrained based on U-Net. They achieved an accuracy of 97% using the ADNI dataset. Andrea [17] developed an automatic deep-ensemble approach for AD classification. They used MRI and fMRI images from the Kaggle, OASIS, and ADNI datasets. They evaluated AlexNet, ResNet-50, ResNet-101, GoogLeNet, and Inception-ResNet-v2 architectures. The proposed approach achieved 98.51% and 98.67% accuracy in binary and multi-class classification. Serkan [17] used different pretrained CNN architecture for the trinary classification of AD. T1-weighted sMRI 2182 images were used from the ADNI database. After data acquisition, preprocessing was performed in three steps. For data analysis, he used DL architectures created with the CNN algorithm. The EfficientNetB0 model achieved the best accuracy of 92.98%.

CNN and deep learning-based approaches have been widely studied as a key methodology for AD diagnosis. However, there are still challenges, such as the MRI image complexity, CNN-based methods that cannot analyze MRI images on the deep structure, the empirical design of DL technologies, limited datasets, time and space complexity, inaccuracy, and large model parameters and hyperparameter optimization.

## 4. Methodology

**Figure 5:**
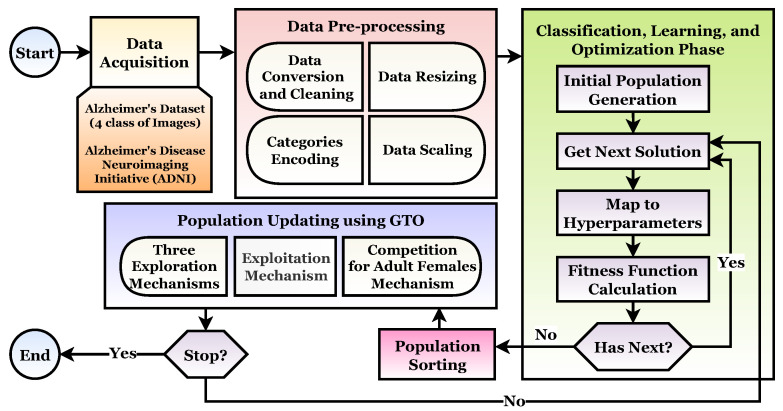
The suggested A${}^{3}$C-TL-GTO framework.

The main objective of this study is to introduce a novel framework for automatic and accurate classification of Alzheimer’s based on MRI images with the help of transfer learning and an Artificial Gorilla Troops Optimizer (GTO). The framework is called A${}^{3}$C-TL-GTO. Figure 5 depicts the different framework stages. The stages and processes will be discussed in the next subsections.

### 4.1. Data Acquisition

The datasets can be retrieved from different sources, such as online repositories. The current study retrieves the datasets from Kaggle and IDA (Image and Data Archive by LONI). In addition, the experiments are performed on two datasets named Alzheimer’s Dataset (4 class of Images) and Alzheimer’s Disease Neuroimaging Initiative (ADNI).

Alzheimer’s Dataset (4 class of Images): This dataset consists of MRI images that are hand-collected from different verified websites [42]. It is partitioned into four classes: Mild Demented, Moderate Demented, Non-Demented, and Very Mild Demented. It consists of 6400 images. The dataset can be retrieved from [42].

Alzheimer’s Disease Neuroimaging Initiative (ADNI): The DICOM data is downloaded from LONI. The current study focused on the MRI T2-weighted axial cases. The data are partitioned into three classes, AD (Alzheimer), NC (Normal Cohort), and MCI (Mild Cognitive Impairment), and organically counted, 17,976, 138,105, and 70,076, respectively [43]. The dataset can be retrieved from (accessed on 1 February 2022) http://adni.loni.usc.edu/ and https://ida.loni.usc.edu/.

**Figure 6:**
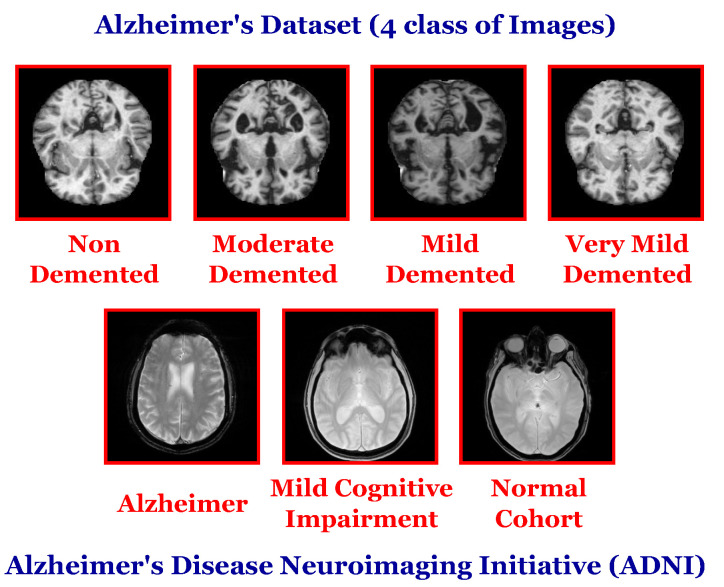
Samples from each used dataset in the current study.

Figure 6 shows samples from each dataset. It shows the “Alzheimer’s Dataset (4 class of Images)” dataset with its four categories in the first row and the “Alzheimer’s Disease Neuroimaging Initiative (ADNI)” dataset with its three categories in the second row.

### 4.2. Data Preprocessing

The second stage focuses on preprocessing the datasets by applying four processes. They are data conversion and cleaning, data resizing, data scaling, and train-to-test splitting.

#### 4.2.1. Data Conversion and Cleaning

**Figure 7:**
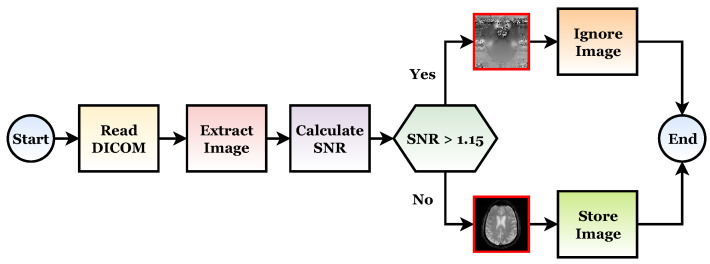
The data conversion and cleaning steps applied on the ADNI dataset.

The ADNI dataset is subjected to the cleaning process. It means that the noisy images are ignored, as shown in Figure 7. In this process, the DICOM records are converted to images, the SNR values are calculated, and the noisy images are removed using a signal-to-noise (SNR) threshold of 1.15.

#### 4.2.2. Data Resizing

The images in the target dataset have various dimensions; hence, equalizing their dimensions (i.e., width and height) is required. The current study uses the size of (128,128,3) using the bicubic interpolation in the RGB color mode.

#### 4.2.3. Categories Encoding

The categories are encoded and converted to numeric values. This process is run on the two used datasets. For example, the ADNI categories (i.e., NC, MCI, and AD) are converted to [0,1,2].

#### 4.2.4. Data Scaling

This study uses 4 image scaling techniques which are: (1) normalization (Equation (1)), (2) standardization (Equation (2)), (3) min-max scaling (Equation (3)), and (4) max-abs scaling (Equation (4)).

(1) $$X_{\mathrm{output}}=\frac{X}{\max\left(X\right)}$$

(2) $$X_{\mathrm{output}}=\frac{X-\mu }{\sigma }$$

(3) $$X_{\mathrm{output}}=\frac{X-\min\left(X\right)}{\max\left(X\right)-\min\left(X\right)}$$

(4) $$X_{\mathrm{output}}=\frac{X}{\left|\max\left(X\right)\right|}$$

where *X* is the input image, $X_{\mathrm{output}}$ is the scaled image, μ is the image mean, and σ is the image standard deviation.

#### 4.2.5. Train-To-Test Splitting

The two used datasets are split into training, testing, and validation subsets. The dataset is partitioned into training (and validation) with 85% images to testing with 15% images.

#### 4.2.6. Dataset Balancing

More records in one category than another, then leads the model to learn, extracting the features from the model with the highest instances better than the others. Hence, data balancing is required to overcome that issue. The current study balances the datasets during the training process using data augmentation techniques that can be applied using different techniques, including GANs [44].

### 4.3. Classification, Learning, and Optimization Phase

After preprocessing the datasets and generating the initial population, the learning phase comes in. This phase utilizes the GTO metaheuristic optimizer to optimize the different transfer learning hyperparameters, such as the appliance of data augmentation and batch size. The followed approach is to find the best hyperparameters configurations for each used pretrained transfer learning model. This stage utilizes three processes. They are summarized in Algorithm 1 and in Figure 5. As presented in it, the first process runs only once, while the other two processes run repeatedly for a number of iterations $T_{max}$.
**Algorithm 1:**The hyperparameters optimization overall process in short.
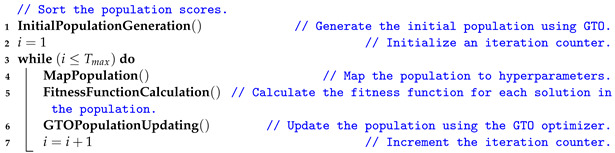


#### 4.3.1. Initial Population Generation

The population is randomly generated once at the beginning of the optimization processes. The number of solutions in the population pack is set to $N_{max}$. Each solution is a vector with a size of 1×D where each element in the solution ∈[0,1]. The value of *D* is determined according to the number of hyperparameters in the current study. It is set to 16. Equation (5) shows the population initialization process.

(5) $$X=rand\times \left(UB-LB\right)+LB$$

where *X* denotes the whole population solutions matrix, LB is the lower boundaries vector, UB is the upper boundaries vector, and rand is a random values vector ∈[0,1].

#### 4.3.2. Fitness Function Calculation

In the current step, the fitness function score is evaluated for each solution. As described earlier, each solution consists of random floating-point numbers ∈[0,1]. Hence, it is required to convert (i.e., map) them to the corresponding hyperparameters as defined in Table 1.

How to apply the mapping technique? To recognize the working mechanism of this mapping process, let us assume that we need to map the batch size (i.e., the second element) from the solution cell to a corresponding hyperparameter. It is required first to determine the allowed batch sizes range to select from. The current study utilizes the “4→48(step=4)” range. Hence, there are 12 possibilities. With a simple calculation (Equation 6), the possibility can be determined. For example, if the random numeric value is 0.75 and there are 12 possibilities, then the index is 9 (i.e., the batch size value of 36). It is worth noting that ranges of each hyperparameter are presented in Table 2.

(6) $$\mathrm{Range}\;\mathrm{Index}=\left\lceil \mathrm{solution}\left[index\right]\times \mathrm{Length}\left(\mathrm{ranges}[index]\right)\right\rceil$$

After mapping each element in the solution to the corresponding hyperparameter, the target pretrained transfer learning model is compiled with the hyperparameters. DenseNet201, MobileNet, MobileNetV2, MobileNetV3Small, MobileNetV3Large, VGG16, VGG19, and Xception with the “ImageNet” pretrained weights are the utilized pretrained transfer learning CNN models. Each pretrained transfer learning CNN model will begin the learning process on the split subsets for a number of epochs that is set to 5 in the current study. To validate its generalization, the pretrained transfer learning CNN model is evaluated on the entire entered dataset.

The different utilized performance metrics in the current study are: Accuracy, F1-score, Precision, Recall (or Sensitivity), Specificity, Area Under Curve (AUC), Intersection over Union (IoU), Dice, Cosine Similarity, Youden Index, Negative Predictive Value (NPV), Matthews Correlation Coefficient (MCC), FBeta, False Negative Rate (FNR), False Discovery Rate (FDR), Fallout, Categorical Crossentropy, Kullback Leibler Divergence (KLD), Categorical Hinge, Hinge, Squared Hinge, Poisson, Logcosh Error, Mean Absolute Error (MAE), Mean IoU, Mean Squared Error (MSE), Mean Squared Logarithmic Error, and Root Mean Squared Error (RMSE).

#### 4.3.3. Population Updating

In terms of fitness scores, the population is arranged in descending order. As a result, the best solution is at the top and the worst solution is at the bottom. This process is crucial to determine $X_{best}^{t}$ and $X_{worst}^{t}$ in the case of them being required in the population updating process. The current study utilizes the GTO metaheuristic optimizer to determine the best hyperparameters for each CNN model.

The GTO works on the (1) three exploration mechanisms, (2) an exploitation mechanism, and (3) a competition for adult females mechanism. Equation (7) represents expanded exploration process, Equation (8) represents the exploitation mechanism, and Equation (9) represents the competition for adult females mechanism.

(7) $$X_{GTO1}\left(t+1\right)=\left\{\begin{array}{cc} \left(UB-LB\right)\times r_{1}+LB, & \mathrm{if}\;(rand<p)\\ \left(r_{2}-C\right)\times X_{r}\left(t\right)+L\times H, & \mathrm{if}\;(rand\geq 0.5)\\ X\left(i\right)-L\times \left(L\times \left(X\left(t\right)-X_{r}\left(t\right)\right)+r_{3}\times \left(X\left(t\right)-X_{r}\left(t\right)\right)\right), & \mathrm{Otherwise} \end{array}\right.$$

(8) $$X_{GTO2}\left(t+1\right)=L\times M\times \left(X\left(t\right)-X_{silverback}\right)+X\left(t\right)$$

(9) $$X_{GTO3}\left(t+1\right)=X_{silverback}-\left(X_{silverback}\times Q-X\left(t\right)\times Q\right)\times A$$

where $r_{1}$, ${}_{2}$, and $r_{3}$ are three random values, $X_{r}\left(t\right)$ is a random solution from the population, $X_{silverback}$ is the silverback gorilla position vector (i.e., best solution), *Q* simulates the impact force, and *A* is the coefficient vector to determine violence degree in conflicts.

### 4.4. The Suggested A${}^{3}$C-TL-GTO Framework Pseudocode

The steps are iteratively computed for a number of iterations $T_{max}$. After completing the learning iterations, the best combination can be used in any further analysis. Algorithm 2 summarizes the proposed overall classification, learning, and hyperparameters optimization approach.
**Algorithm 2:**The suggested A${}^{3}$C-TL-GTO framework pseudocode.
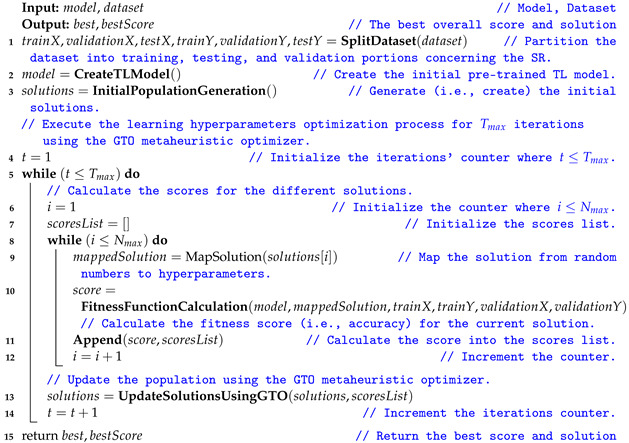


## 5. Experiments and Discussions

### 5.1. Experiments Configurations

The configurations of the experiments performed in this study are described in Table 2.

**Table 2:** The common experiments configurations.

Configuration	Specifications
Apply Dataset Shuffling?	Yes (Random)
Input Image Size	(128×128×3)
Hyperparameters Metaheuristic Optimizer	Artificial Gorilla Troops Optimizer (GTO)
Train Split Ratio	85% to 15% (i.e., 85% for training (and validation) and 15% for testing)
Size of Population	10
Number of Iterations	10
Number of Epochs	5
Output Activation Function	SoftMax
Pretrained Models	DenseNet201, MobileNet, MobileNetV2, MobileNetV3Small, MobileNetV3Large, VGG16, VGG19, and Xception
Pretrained Parameters Initializers	ImageNet
Losses Range	Categorical Crossentropy, Categorical Hinge, KLDivergence, Poisson, Squared Hinge, and Hinge
Parameters Optimizers Range	Adam, NAdam, AdaGrad, AdaDelta, AdaMax, RMSProp, SGD, Ftrl, SGD Nesterov, RMSProp Centered, and Adam AMSGrad
Dropout Range	[0→0.6]
Batch Size Range	4→48(step=4)
Pretrained Model Learn Ratio Range	1→100(step=1)
Scaling Techniques	Normalize, Standard, Min-Max, and Max-Abs
Apply Data Augmentation (DA)	[Yes,No]
DA Rotation Range	$0^{\circ }\to 45^{\circ }\;\left(\mathrm{step}=1^{\circ }\right)$
DA Width Shift Range	[0→0.25]
DA Height Shift Range	[0→0.25]
DA Shear Range	[0→0.25]
DA Zoom Range	[0→0.25]
DA Horizontal Flip Range	[Yes,No]
DA Vertical Flip Range	[Yes,No]
DA Brightness Range	[0.5→2.0]
Scripting Language	Python
Python Major Packages	Tensorflow, Keras, Pydicom, NumPy, OpenCV, and Matplotlib
Working Environment	Google Colab with GPU (i.e., Intel(R) Xeon(R) CPU @ 2.00 GHz, Tesla T4 16 GB GPU, CUDA v.11.2, and 12 GB RAM)

### 5.2. The “Alzheimer’s Dataset (4 Class of Images)” Experiments

**Figure 8:**
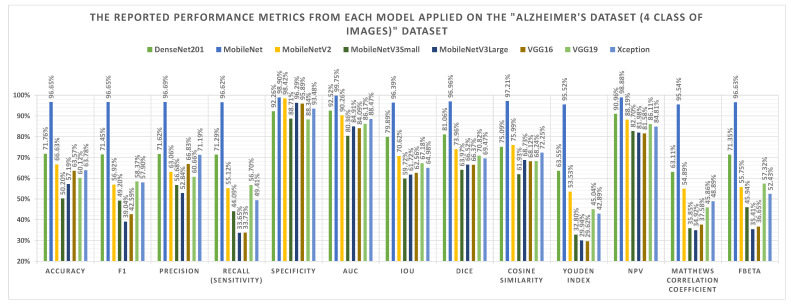
Graphical summary of the performance metrics of the “Alzheimer’s Dataset (4 class of Images)” dataset.

**Figure 9:**
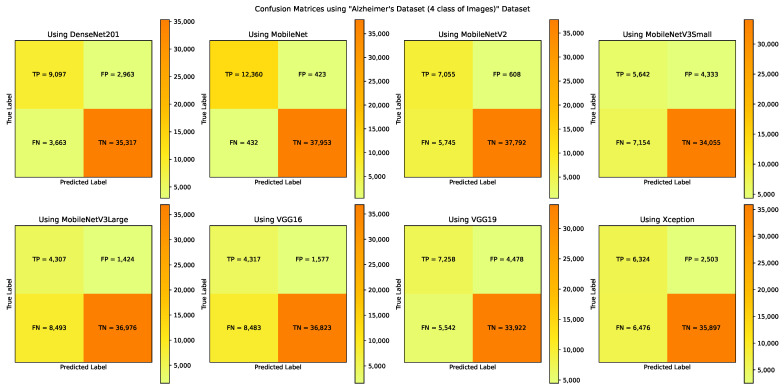
The confusion matrices using the “Alzheimer’s Dataset (4 class of Images)” dataset.

The A${}^{3}$C-TL-GTO framework stages are run on the “Alzheimer’s Dataset (4 class of Images)” dataset. Table 3 reports the confusion matrix (i.e., TP, TN, FP, and FN) for each pretrained CNN model. From Table 3, different performance metrics can be reported, as shown in Table 4. Table 5 reports the corresponding best hyperparameters produced that lead to the reported results. The “Categorical Crossentropy” is the recommended loss function from five models. The “SGD” is the recommended parameters’ optimizer from three models. Applying data augmentation to balance and increase the diversity of the images during the training process is recommended by six models. Figure 8 summarizes the performance metrics graphically. The x-axis shows the metrics, while the y-axis shows the scores. It shows that the “MobileNet” pretrained CNN model reports the highest performance metrics. Figure 9 shows the confusion matrices for the used models.

### 5.3. The “Alzheimer’s Disease Neuroimaging Initiative (ADNI)” Experiments

**Figure 10:**
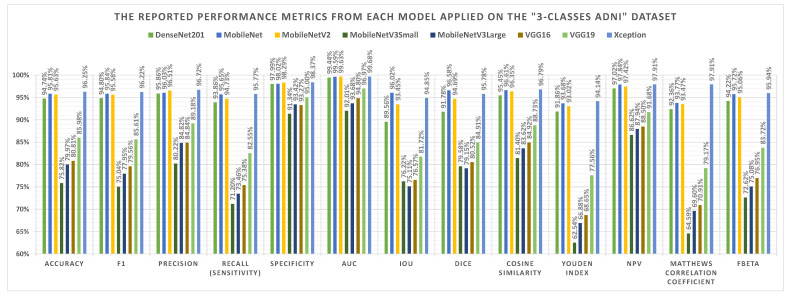
Graphical summary of the performance metrics of the “Alzheimer’s Disease Neuroimaging Initiative (ADNI)” dataset.

**Figure 11:**
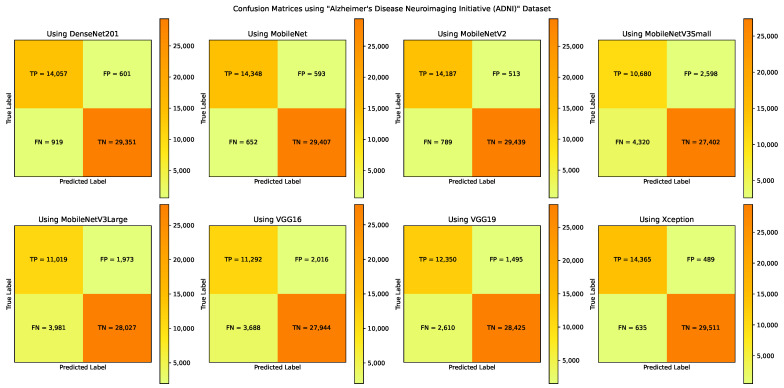
The confusion matrices using the “Alzheimer’s Disease Neuroimaging Initiative (ADNI)” dataset.

The A${}^{3}$C-TL-GTO framework stages are run on the “Alzheimer’s Disease Neuroimaging Initiative (ADNI)” dataset. Table 6 reports the confusion matrix (i.e., TP, TN, FP, and FN) for each pretrained CNN model. From Table 6, different performance metrics can be reported, as shown in Table 7. Table 8 reports the corresponding best hyperparameters produced that lead to the reported results. The “KLDivergence” is the recommended loss function from six models. The “AdaGrad” and “AdaMax” are the recommended parameters’ optimizers from three models each. Applying data augmentation to balance and increase the diversity of the images during the training process is recommended by seven models. Figure 10 summarizes the performance metrics graphically. The x-axis shows the metrics, while the y-axis shows the scores. It shows that the “Xception” pretrained CNN model reports the highest performance metrics. Figure 11 shows the confusion matrices for the used models.

**Figure 12:**
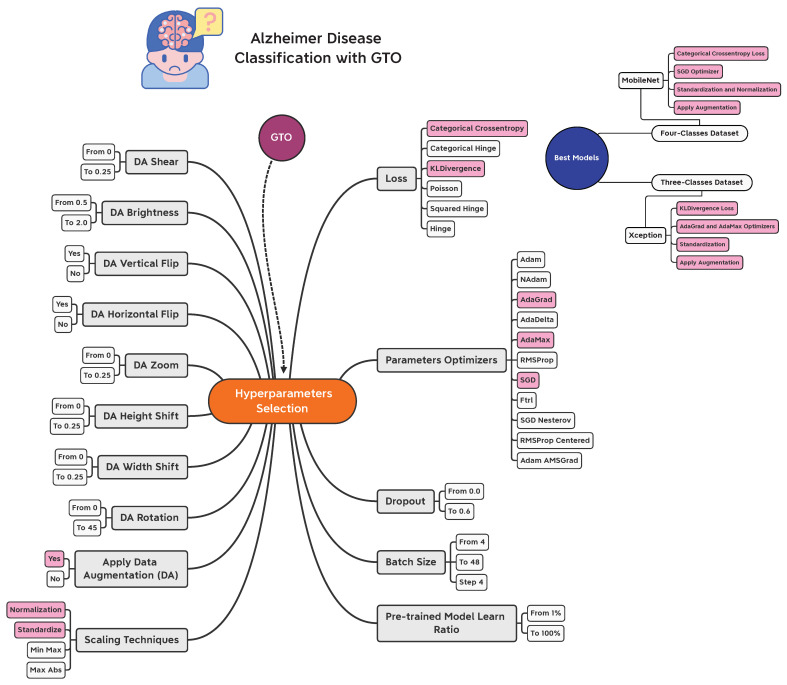
Graphical summary of the performed work in the current study concerning the hyperparameters selection process.

Figure 12 presents a graphical summary of the performed work in the current study concerning the hyperparameters selection process. The best models are added at the right of the figure. The different hyperparameters are added in a gray color, while the best hyperparameters are added in a different color.

### 5.4. The Proposed Approach and Related Studies Comparison

A comparison between the suggested A${}^{3}$C-TL-GTO framework and other related state-of-the-art studies is conducted in Table 9. It is clear that the A${}^{3}$C-TL-GTO framework outperforms most of the related studies. One of the main objectives of the suggested approach is to design a general framework that utilizes the pretrained CNN model and hyperparameters tuning using metaheuristic optimizers. In other words, the framework is adaptable to the metaheuristic optimizer and the used datasets. Hence, in comparison with the related studies, the systems are compared as black boxes. One of the main advantages of the suggested framework is that it is not sensitive to the datasets and their outliers.

## 6. Conclusions

With the rapid growth of artificial intelligence, computer vision has become increasingly helpful in identifying Alzheimer’s disease. In recent years, deep learning technology has increasingly dominated medical imaging and has been successfully used to automate AD detection by analyzing medical pictures. A deep network model based on transfer learning, which Gorilla Troops optimizes, has been developed to aid in the classification of Alzheimer’s disease patients for early diagnosis. In the present study, an empirical quantitative framework for automatic and accurate Alzheimer’s classification is proposed and evaluated using multi-class MRI datasets. The convolutional neural network (CNN) performance is primarily affected by the hyperparameters selected and the dataset used. The proposed framework reduces the bias and variability of the preprocessing steps and optimization hyperparameters to the classifier model and dataset utilized. Specifically, the proposed framework comprises CNN, transfer learning (TL), and the Gorilla Troops Optimizer (GTO) for optimizing parameters and hyperparameters. The transfer learning hyperparameters are optimized using the GTO natural-inspired optimizers. The ADNI dataset, an online dataset on Alzheimer’s disease, is used to obtain the brain’s magnetic resonance (MR) pictures. When all models are compared, MobileNet and Xception achieved a top accuracy of 96.65% and 96.25%, respectively.

## Figures and Tables

**Table 1:** The solution indexing with the hyperparameters definitions.

Element Index	Hyperparameter Definition
1	Loss function
2	Batch size
3	Dropout ratio
4	TL learning ratio
5	Weights (i.e., parameters) optimizer
6	Dimension scaling technique
7	Apply data augmentation or not
8	Rotation value (in case of data augmentation is applied)
9	Width shift value (in case of data augmentation is applied)
10	Height shift value (in case of data augmentation is applied)
11	Shear value (in case of data augmentation is applied)
12	Zoom value (in case of data augmentation is applied)
13	Horizontal flipping flag (in case of data augmentation is applied)
14	Vertical flipping flag (in case of data augmentation is applied)
15	Brightness changing range (in case of data augmentation is applied)

**Table 3:** The confusion matrix (i.e., TP, TN, FP, and FN) for each pretrained CNN model using the “Alzheimer’s Dataset (4 class of Images)” dataset.

Model Name	DenseNet201	MobileNet	MobileNetV2	MobileNetV3Small	MobileNetV3Large	VGG16	VGG19	Xception	Best Score	Worst Score
TP	9097	12,360	7055	5642	4307	4317	7258	6324	12,360	4307
TN	35,317	37,953	37,792	34,055	36,976	36,823	33,922	35,897	37,953	33,922
FP	2963	423	608	4333	1424	1577	4478	2503	423	4478
FN	3663	432	5745	7154	8493	8483	5542	6476	432	8493

**Table 4:** The best performance metrics reported by the “Alzheimer’s Dataset (4 class of Images)” dataset.

Model Name	DenseNet201	MobileNet	MobileNetV2	MobileNetV3Small	MobileNetV3Large	VGG16	VGG19	Xception	Best
Loss	0.891	0.094	0.702	1.079	0.926	0.795	1.102	0.798	0.094
Accuracy	71.76%	96.65%	66.63%	50.20%	57.19%	63.57%	60.12%	63.78%	96.65%
F1	71.45%	96.65%	56.92%	49.20%	39.04%	42.59%	58.37%	57.90%	96.65%
Precision	71.62%	96.69%	63.06%	56.68%	52.84%	66.83%	60.66%	71.19%	96.69%
Recall (Sensitivity)	71.29%	96.62%	55.12%	44.09%	33.65%	33.73%	56.70%	49.41%	96.62%
Specificity	92.26%	98.90%	98.42%	88.71%	96.29%	95.89%	88.34%	93.48%	98.90%
AUC*	92.52%	99.75%	90.26%	80.36%	84.91%	84.09%	86.17%	88.47%	99.75%
IoU*	79.89%	96.39%	70.62%	59.72%	61.72%	62.56%	67.18%	64.98%	96.39%
Dice	81.06%	96.96%	73.96%	63.97%	66.52%	66.37%	70.82%	69.47%	96.96%
Cosine Similarity	75.09%	97.21%	75.99%	61.93%	68.73%	68.12%	68.24%	72.25%	97.21%
Youden Index	63.55%	95.52%	53.53%	32.80%	29.94%	29.62%	45.04%	42.89%	95.52%
NPV *	90.96%	98.88%	88.19%	82.70%	81.98%	81.58%	86.11%	84.81%	98.88%
MCC *	63.11%	95.54%	54.89%	35.85%	34.92%	37.58%	45.86%	48.89%	95.54%
FBeta	71.35%	96.63%	55.75%	45.94%	35.41%	36.65%	57.32%	52.43%	96.63%
FNR *	0.287	0.034	0.449	0.559	0.664	0.663	0.433	0.506	0.034
FDR *	0.284	0.033	0.129	0.433	0.169	0.268	0.393	0.288	0.033
Fallout	0.077	0.011	0.016	0.113	0.037	0.041	0.117	0.065	0.011
CC *	0.891	0.094	0.702	1.079	0.926	1.086	0.973	0.798	0.094
KLD *	0.891	0.094	0.702	1.079	0.924	1.086	0.973	0.798	0.094
Categorical Hinge	0.506	0.090	0.593	0.926	0.852	0.795	0.802	0.776	0.090
Hinge	0.892	0.773	0.945	1.020	1.001	1.002	0.969	0.979	0.773
Squared Hinge	0.993	0.786	1.039	1.173	1.129	1.138	1.102	1.096	0.786
Poisson	0.473	0.274	0.426	0.520	0.481	0.521	0.493	0.449	0.274
Logcosh Error	0.045	0.006	0.044	0.071	0.060	0.063	0.061	0.055	0.006
MAE *	0.142	0.023	0.195	0.270	0.251	0.252	0.219	0.229	0.023
Mean IoU	0.389	0.424	0.375	0.375	0.375	0.375	0.375	0.375	0.375
MSE *	0.101	0.013	0.094	0.153	0.128	0.135	0.133	0.117	0.013
MSLE *	0.051	0.006	0.046	0.075	0.063	0.066	0.066	0.057	0.006
RMSE *	0.318	0.113	0.306	0.391	0.358	0.368	0.365	0.341	0.113

* AUC: Area Under Curve, IoU: Intersection over Union, NPV: Negative Predictive Value, MCC: Matthews Correlation Coefficient, FNR: False Negative Rate, FDR: False Discovery Rate, CC: Categorical Crossentropy, KLD: Kullback Leibler Divergence, MAE: Mean Absolute Error, MSE: Mean Squared Error, MSLE: Mean Squared Logarithmic Error, RMSE: Root Mean Squared Error.

**Table 5:** The best hyperparameters for each pretrained CNN model using the “Alzheimer’s Dataset (4 class of Images)” dataset.

Model Name	DenseNet201	MobileNet	MobileNetV2	MobileNetV3Small	MobileNetV3Large	VGG16	VGG19	Xception
Loss	Categorical Crossentropy	Categorical Crossentropy	Categorical Crossentropy	Categorical Crossentropy	Categorical Crossentropy	Categorical Hinge	Squared Hinge	Categorical Crossentropy
Batch Size	44	12	20	28	4	16	40	40
Dropout	0.13	0.24	0.6	0.52	0.33	0.05	0.06	0.22
TL Learn Ratio	97	65	74	54	92	32	52	6
Optimizer	SGD	SGD	SGD Nesterov	NAdam	RMSProp	AdaGrad	SGD	NAdam
Scaling Technique	Standardization	Min-Max	Standardization	Normalization	Max-Abs	Normalization	Standardization	Normalization
Apply Augmentation	Yes	No	Yes	Yes	Yes	Yes	Yes	No
Rotation Range	13	N/A	5	33	4	4	21	N/A
Width Shift Range	0.05	N/A	0.25	0.05	0.17	0.07	0.03	N/A
Height Shift Range	0.07	N/A	0.23	0	0.03	0.02	0.13	N/A
Shear Range	0.2	N/A	0	0.1	0.02	0	0.07	N/A
Zoom Range	0.17	N/A	0	0.1	0.02	0.02	0.23	N/A
Horizontal Flip	Yes	N/A	No	No	Yes	Yes	No	N/A
Vertical Flip	Yes	N/A	Yes	Yes	No	Yes	Yes	N/A
Brightness Range	0.54–0.8	N/A	0.5–1.51	1.83–2.0	0.92–1.53	0.63–0.82	0.81–1.93	N/A

**Table 6:** The confusion matrix (i.e., TP, TN, FP, and FN) for each pretrained CNN model using the “Alzheimer’s Disease Neuroimaging Initiative (ADNI)” dataset.

Model Name	DenseNet201	MobileNet	MobileNetV2	MobileNetV3Small	MobileNetV3Large	VGG16	VGG19	Xception	Best Score	Worst Score
TP	14,057	14,348	14,187	10,680	11,019	11,292	12,350	14,365	14,365	10,680
TN	29,351	29,407	29,439	27,402	28,027	27,944	28,425	29,511	29,511	27,402
FP	601	593	513	2598	1973	2016	1495	489	489	2598
FN	919	652	789	4320	3981	3688	2610	635	635	4320

**Table 7:** The best performance metrics reported by the “Alzheimer’s Disease Neuroimaging Initiative (ADNI)” dataset.

Model Name	DenseNet201	MobileNet	MobileNetV2	MobileNetV3Small	MobileNetV3Large	VGG16	VGG19	Xception	Best
Loss	0.171	0.112	0.122	0.544	0.495	0.448	0.347	0.106	0.106
Accuracy	94.74%	95.81%	95.63%	75.82%	79.97%	80.81%	85.98%	96.25%	96.25%
F1	94.80%	95.84%	95.58%	75.04%	77.95%	79.56%	85.61%	96.22%	96.22%
Precision	95.86%	96.03%	96.51%	80.22%	84.82%	84.84%	89.18%	96.72%	96.72%
Recall (Sensitivity)	93.86%	95.65%	94.73%	71.20%	73.46%	75.38%	82.55%	95.77%	95.77%
Specificity	97.99%	98.02%	98.29%	91.34%	93.42%	93.27%	95.00%	98.37%	98.37%
AUC *	99.44%	99.59%	99.63%	92.01%	93.68%	94.80%	96.97%	99.68%	99.68%
IoU *	89.56%	96.02%	93.45%	76.22%	75.11%	76.57%	81.72%	94.85%	96.02%
Dice	91.78%	96.58%	94.69%	79.58%	79.15%	80.52%	84.91%	95.78%	96.58%
Cosine Similarity	95.45%	96.61%	96.35%	81.40%	83.62%	84.92%	88.73%	96.79%	96.79%
Youden Index	91.86%	93.68%	93.02%	62.54%	66.88%	68.65%	77.56%	94.14%	94.14%
NPV *	97.02%	97.84%	97.42%	86.62%	87.94%	88.50%	91.68%	97.91%	97.91%
MCC *	92.36%	93.77%	93.47%	64.59%	69.60%	70.91%	79.17%	97.91%	97.91%
FBeta	94.22%	95.72%	95.06%	72.62%	75.08%	76.95%	83.72%	95.94%	95.94%
FNR *	0.061	0.043	0.053	0.288	0.265	0.246	0.174	0.042	0.042
FDR *	0.041	0.040	0.035	0.198	0.152	0.152	0.108	0.033	0.033
Fallout	0.020	0.020	0.017	0.087	0.066	0.067	0.050	0.016	0.016
CC *	0.171	0.112	0.122	0.544	0.495	0.448	0.347	0.106	0.106
KLD *	0.171	0.112	0.122	0.544	0.495	0.448	0.347	0.106	0.106
Categorical Hinge	0.225	0.099	0.147	0.545	0.553	0.529	0.409	0.120	0.099
Hinge	0.749	0.701	0.720	0.871	0.875	0.861	0.818	0.709	0.701
Squared Hinge	0.777	0.721	0.742	0.976	0.970	0.949	0.884	0.728	0.721
Poisson	0.390	0.371	0.374	0.515	0.498	0.483	0.449	0.369	0.369
Logcosh Error	0.013	0.009	0.010	0.049	0.045	0.041	0.031	0.009	0.009
MAE *	0.082	0.034	0.053	0.204	0.209	0.195	0.151	0.042	0.034
Mean IoU	0.333	0.414	0.337	0.333	0.333	0.333	0.333	0.347	0.333
MSE *	0.028	0.020	0.022	0.105	0.095	0.088	0.066	0.019	0.019
MSLE *	0.014	0.010	0.011	0.052	0.047	0.043	0.033	0.009	0.009
RMSE *	0.168	0.142	0.148	0.324	0.308	0.296	0.257	0.139	0.139

* AUC: Area Under Curve, IoU: Intersection over Union, NPV: Negative Predictive Value, MCC: Matthews Correlation Coefficient, FNR: False Negative Rate, FDR: False Discovery Rate, CC: Categorical Crossentropy, KLD: Kullback Leibler Divergence, MAE: Mean Absolute Error, MSE: Mean Squared Error, MSLE: Mean Squared Logarithmic Error, RMSE: Root Mean Squared Error.

**Table 8:** The best hyperparameters for each pretrained CNN model using the “Alzheimer’s Disease Neuroimaging Initiative (ADNI)” dataset.

Model Name	DenseNet201	MobileNet	MobileNetV2	MobileNetV3Small	MobileNetV3Large	VGG16	VGG19	Xception
Loss	Categorical Crossentropy	Categorical Crossentropy	KLDivergence	KLDivergence	KLDivergence	KLDivergence	KLDivergence	KLDivergence
Batch Size	32	40	36	20	12	28	44	40
Dropout	0.1	0.23	0.3	0.34	0.13	0.11	0.02	0.3
TL Learn Ratio	37	28	41	53	94	71	99	39
Optimizer	AdaGrad	AdaMax	SGD Nesterov	AdaMax	AdaGrad	AdaGrad	SGD Nesterov	AdaMax
Scaling Technique	Standardization	Standardization	Min-Max	Standardization	Normalization	Min-Max	Standardization	Max-Abs
Apply Augmentation	No	Yes	Yes	Yes	Yes	Yes	Yes	Yes
Rotation Range	N/A	44	12	15	28	3	12	27
Width Shift Range	N/A	0.2	0.17	0.13	0.09	0.16	0.22	0.17
Height Shift Range	N/A	0.23	0.16	0.08	0.25	0.23	0.23	0.12
Shear Range	N/A	0.07	0.17	0.06	0.25	0.23	0.08	0.21
Zoom Range	N/A	0.22	0.19	0.14	0.06	0.03	0.08	0.1
Horizontal Flip	N/A	Yes	Yes	Yes	No	No	No	Yes
Vertical Flip	N/A	No	Yes	Yes	Yes	Yes	Yes	No
Brightness Range	N/A	0.56–0.68	1.09–1.48	0.85–1.67	1.48–2.0	0.52–1.34	1.23–1.51	0.53–1.82

**Table 9:** Comparison between the suggested approach and related studies.

Study	Year	Approach	Best Metric(s)
Islam and Zhang [33]	2017	DL	73.75% Accuracy
Zhang et al. [34]	2019	Voxel-based Morphometry	96% Accuracy
Martinez et al. [35]	2019	DL + Autoencoders	95% Accuracy
Saratxaga et al. [2]	2021	DL	93% Balanced Accuracy
Raees et al. [36]	2021	DL	90% Accuracy
Buvaneswari et al. [37]	2021	DL	95% Accuracy
Katabathula et al. [38]	2021	3D DL	92.5% Accuracy
Current Study (A${}^{3}$C-TL-GTO)	2022	Hybrid (GTO + DL)	96.65% Accuracy for “Alzheimer’s Dataset (4 class of Images)” and 96.25% Accuracy “Alzheimer’s Disease Neuroimaging Initiative (ADNI)”

## Data Availability

Data available upon request.
